# Propensity scores in the presence of effect modification: A case study using the comparison of mortality on hemodialysis versus peritoneal dialysis

**DOI:** 10.1186/1742-7622-7-1

**Published:** 2010-05-11

**Authors:** Ylian S Liem, John B Wong, MG Myriam Hunink, Frank Th de Charro, Wolfgang C Winkelmayer

**Affiliations:** 1 Program for the Assessment of Radiological Technology (ART Program), Department of Epidemiology & Biostatistics and the Department of Radiology, Erasmus University Medical Center Rotterdam, Dr. Molewaterplein 50, 3015 GE Rotterdam, the Netherlands; 2 Department of Internal Medicine, Erasmus University Medical Center Rotterdam, Dr. Molewaterplein 50, 3015 GE Rotterdam, the Netherlands; 3 Division of Clinical Decision Making, Informatics and Telemedicine, Department of Medicine, Tufts Medical Center, Tufts University School of Medicine, 800 Washington Street, #302, Boston, MA 02111, USA; 4 Department of Health Policy and Management, Harvard School of Public Health, Kresge 4th Floor, 677 Huntington Avenue, Boston, Massachusetts 02115, USA; 5 Dutch End Stage Renal Disease Registry RENINE, Postbus 2304, 2301 CH Leiden, The Netherlands; 6 Division of Nephrology, Department of Medicine, Stanford University School of Medicine, 780 Welch Road, Suite 106, Palo Alto, CA 94304, USA

## Abstract

**Purpose:**

To control for confounding bias from non-random treatment assignment in observational data, both traditional multivariable models and more recently propensity score approaches have been applied. Our aim was to compare a propensity score-stratified model with a traditional multivariable-adjusted model, specifically in estimating survival of hemodialysis (HD) versus peritoneal dialysis (PD) patients.

**Methods:**

Using the Dutch End-Stage Renal Disease Registry, we constructed a propensity score, predicting PD assignment from age, gender, primary renal disease, center of dialysis, and year of first renal replacement therapy. We developed two Cox proportional hazards regression models to estimate survival on PD relative to HD, a propensity score-stratified model stratifying on the propensity score and a multivariable-adjusted model, and tested several interaction terms in both models.

**Results:**

The propensity score performed well: it showed a reasonable fit, had a good c-statistic, calibrated well and balanced the covariates. The main-effects multivariable-adjusted model and the propensity score-stratified univariable Cox model resulted in similar relative mortality risk estimates of PD compared with HD (0.99 and 0.97, respectively) with fewer significant covariates in the propensity model. After introducing the missing interaction variables for effect modification in both models, the mortality risk estimates for both main effects and interactions remained comparable, but the propensity score model had nearly as many covariates because of the additional interaction variables.

**Conclusion:**

Although the propensity score performed well, it did not alter the treatment effect in the outcome model and lost its advantage of parsimony in the presence of effect modification.

## Introduction

Using observational data to compare outcomes associated with different treatments may result in biased estimates because of non-random treatment assignment. To correct for variables that may confound an association, the traditional approach is to apply multivariable-adjusted modeling, but in recent years, use of propensity scores has become increasingly popular [1]. The concept of a multivariate confounder score was first introduced by Miettinen in 1976 [2], but the formal concept of propensity scores to estimate causal effects in observational studies was first described by Rosenbaum and Rubin [3]. A propensity score is a conditional probability of assignment to a particular treatment given a vector of baseline covariates. Except for unmeasured potential confounding factors, two patients having the same propensity score but assigned to different treatments are considered to be equivalent to a random assignment of treatment. Thus, adjustment for the propensity score in the outcome model can balance the observed and included covariates and remove bias that may arise due to these confounders. This adjustment can be accomplished by either 1) selecting matched pairs of patients each on a different treatment arm, but with similar propensity scores, 2) stratifying the sample on the propensity score, calculating the treatment effect within strata and then pooling the strata-specific treatment effect estimates, or 3) including the propensity score itself as a covariate in the outcome model.

Several advantages of propensity score-stratified versus traditional multivariable-adjusted modeling have been suggested. The propensity model does not need to be parsimonious and easy to understand because it is not the focus of the study [4]. Furthermore, the propensity score enables a direct estimation of comparability of the treatment groups by assessing the covariate balance between groups. Inability to balance confounders alerts investigators that the treatment groups are not sufficiently overlapping with respect to these confounders and that selection bias may not be resolvable [4]. Traditional multivariable regression modeling will not detect this directly.

Patients with end stage renal disease (ESRD) require renal replacement therapy (RRT). Of all therapeutic options, renal transplantation is generally associated with the highest survival and quality of life. However, due to the shortage of organs, the majority of ESRD patients are treated with renal dialysis. Two main forms of renal dialysis can be distinguished: hemodialysis (HD) and peritoneal dialysis (PD). Many factors influence dialysis treatment assignment: not only the clinical characteristics of a patient, but also patient and physician preference, cultural factors and reimbursement policy decisions may play a role. Therefore, comparison of patient survival on HD and PD is complicated. Because the one randomized controlled trial that has been undertaken to assess survival differences had to be stopped prematurely because of low inclusion rates [5], observational studies have to be relied upon to compare survival on HD versus PD. Our aim was to compare a propensity score-stratified model with a traditional multivariable-adjusted model, specifically in estimating survival of hemodialysis (HD) versus peritoneal dialysis (PD) patients to assess the possible advantages of using a propensity score.

## Analysis

### Methods

#### Patients

We included all incident patients who started RRT between January 1^st ^1987 (start of prospective registration) and December 31^st ^2002 from the Dutch End Stage Renal Disease Registry (RENINE). We excluded patients younger than 18 years, patients who underwent RRT for less than 30 days, patients who had more than one episode of recovery of renal function, or who died directly following a period of renal recovery, patients who received a pre-emptive transplantation, patients who died during the first 90 days of renal replacement therapy and patients from centers treating fewer than 20 dialysis patients or fewer than 5 PD patients. The outcome of interest was all-cause mortality, as registered by RENINE. The registry collects information on date and cause of death and verifies its information yearly with all centers [6,7]. From registry data we also determined age and gender of patients, baseline dialysis modality, year of first dialysis, and the center at which dialysis was started. Modality switches among HD, PD, and kidney transplantation over time were also recorded. In the database, primary renal diagnosis was coded according to the classification of the European Renal Association-European Dialysis and Transplantation Association (ERA-EDTA). After examining previously published disease categories and hazard rates in the Dutch registry, we aggregated these into five categories: glomerulonephritis (PRD-GN), hypertension (PRD-HT), renovascular disease (PRD-RVD), diabetes mellitus (PRD-DM) and a category for all other renal diagnoses (PRD-OTH).

#### Statistical modeling

We adopted an intention-to-treat perspective and, as is customary in previously published analyses, considered the dialysis modality on day 91 to be the definite modality. We left-censored survival time for the first 90 days and right-censored at first transplantation or December 31^st ^2002, whichever occurred first. To estimate the independent comparison between PD and HD mortality by controlling for observed potential confounders, we explored two analytical options: a propensity score-adjustment approach and the traditional multivariable-adjustment approach.

The propensity-score approach involved a two-step approach. First, we predicted PD versus HD treatment assignment by constructing a logistic regression model that estimated treatment assignment using all available variables, as well as age-squared, age-cubed, and all possible 2-way interactions between the database-variables. As explained earlier, this model did not need to be parsimonious nor easy to understand, because it was not the focus of the study. The model calculated the expected probability or propensity score of each patient being assigned to PD, accounting for that individual's baseline characteristics. The propensity score was then evaluated for the following criteria: 1) a reasonable Nagelkerke's r^2^-statistic as a measure of fit and a *c*-statistic between 0.65 and 0.85 as a measure of discriminative power, 2) good calibration as measured by the PS-predicted and observed proportion of PD patients within quintiles of the propensity score, and 3) balanced covariates within quintiles of the propensity score [8]. This third criterion is most important for assessing the appropriateness of the PS-model [9]. In the second-step, estimating the effect of treatment assignment on outcome adjusted for the propensity score, we stratified a Cox model containing dialysis modality as the only independent variable on intervals of 0.01 of the propensity score. Alternative techniques to adjust for the propensity score include matching or regression. However, regression is affected by measurement errors in the propensity score [10]. Furthermore, it assumes a linear relationship between the propensity score and the natural logarithm of the hazard. Matching or stratification techniques do not assume such a relationship, but matching entails exclusion of patients because of the unavailability of a match. Stratification on intervals of 0.01 closely resembles matching, but because the number of patients in either exposure group within a stratum may vary, only few patients will need to be excluded. In our analyses, ten strata not containing either HD or PD patients were excluded.

In the alternative multivariable-adjustment approach, the calculation of the relative mortality of PD patients compared with HD patients was conducted by entering observed characteristics as covariates into the survival regression model and thereby adjusting for potential confounders. The first step in this approach was to estimate univariable Cox proportional hazards models for all available variables. Age and year of start of dialysis were entered into the model as continuous variables and all other variables as categorical variables. All statistically significant variables (P < 0.05) from the univariable analyses were introduced into a multivariable main-effects Cox proportional hazards model. From this multivariable model, we explored the significance of a quadratic term (age) and several two-way and three-way interaction terms. We tested for center effects by entering center as a categorical variable into the multivariable model. Finally, we compared the hazard ratios (HR) for mortality with PD versus HD from the propensity score-stratified and the multivariable-adjusted models.

### Results

#### Patients

The RENINE Registry prospectively collected data of 20,687 patients who started RRT between January 1^st ^1987 and December 31^st ^2002. We discarded 4,044 patients that did not meet inclusion criteria. As a result, our final sample included 16,643 patients from 47 centers. Mean age was 59 years (standard deviation, SD: 15.3) and 58.8% were male. Additional descriptive characteristics are shown in Table 1.

**Table 1:** Baseline characteristics of study cohort

	All patients		HD		PD		P-value
Number (%)	16,643		10,841	(65.1)	5,802	(34.9)	
Age (SD) (yr)	59.0	(15.3)	61.8	(14.6)	53.6	(15.0)	<0.001
Female gender (%)	41.2		42.5		38.7		<0.001
Primary renal disease (%)							<0.001
GN	13.7		11.1		18.5		
HT	11.4		11.7		10.8		
RVD	8.7		9.8		6.6		
DM	15.2		14.9		16.0		
Other	51.0		52.5		48.1		
Year of first RRT (%)							<0.001
1987-1990	17.0		18.0		14.9		
1991-1994	23.5		23.2		24.0		
1995-1998	28.6		28.3		29.0		
1999-2002	31.0		30.5		32.0		
Years of follow-up (SD)	2.38	(2.14)	2.42	(2.24)	2.32	(1.95)	0.007

SD = standard deviation, yr = year, GN = glomerulonephritis, HT = hypertension, RVD = renovascular disease, DM = diabetes mellitus, RRT = renal replacement therapy, HD = hemodialysis, PD = peritoneal dialysis

#### Propensity score analysis

The propensity score model containing age, age^2^, age^3^, all other variables and all possible two-way interactions had a Nagelkerke's r^2 ^of 0.240 and a *c*-statistic of 0.752. Leaving all non-significant variables out of the model did not alter these quality indicators substantially.

**Figure 1:**
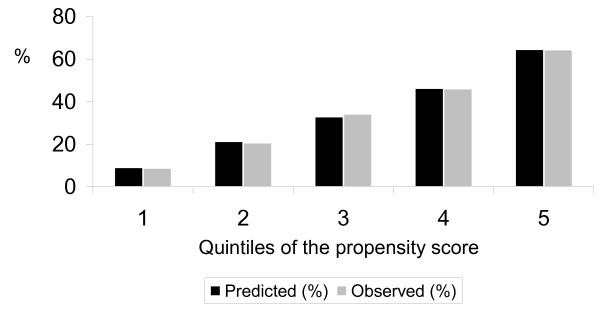
**Mean predicted and observed probability of peritoneal dialysis assignment per quintile of the propensity score**. Predicted (%): probability of assignment to peritoneal dialysis as predicted by the propensity score; Observed (%): actual prevalence of peritoneal dialysis assignment.

The propensity score in quintiles showed good calibration. The mean propensity scores (the probability of receiving PD) were 9.1%, 21.4% 32.9%, 46.3% and 64.6% for each quintile, respectively and were very similar to the actual proportions of patients on peritoneal dialysis in all quintiles (see Figure 1). Furthermore, the propensity score balanced the covariates between the HD and PD groups except for a slight (1.4 year) difference in age within the fifth quintile and in the starting year within the second and the fifth quintile (Table 2). Stratifying the univariable Cox model on 0.01 intervals of the propensity score yielded no difference in mortality risk between PD and HD patients (HR = 0.97; 95%CI 0.92-1.02) (Table 3).

**Table 2:** Baseline characteristics of study cohort, by quintile of propensity score

Quintile		1			2			3			4			5	
	**HD**	**PD**	**p**	**HD**	**PD**	**p**	**HD**	**PD**	**p**	**HD**	**PD**	**p**	**HD**	**PD**	**p**

N	3,046	295		2,643	691		2,157	1,127		1,813	1,548		1,182	2,141	
(%)	(91.2)	(8.8)		(79.3)	(20.7)		(65.7)	(34.3)		(53.9)	(46.1)		(35.6)	(64.4)	

Age (yr)	72.5	72.2	0.66	65.0	65.7	0.16	59.0	58.6	0.38	52.7	52.5	0.19	46.6	45.2	0.003
Male gender (%)	49.0	50.2	0.71	58.2	58.5	0.90	62.8	62.6	0.92	61.0	61.0	0.98	63.0	63.1	0.95
Primary renal disease (%)			0.27			0.74			0.89			0.37			0.08
GN	7.4	4.4		7.3	6.1		11.1	11.4		12.3	14.5		27.3	31.2	
HT	11.2	12.9		12.4	11.7		12.1	11.8		13.0	12.9		8.8	8.2	
RVD	13.0	14.2		11.8	12.7		9.6	8.5		6.5	6.3		3.2	2.7	
DM	14.0	15.9		14.7	14.5		15.4	16.0		14.3	13.0		17.3	18.6	
Other	54.5	52.5		53.8	55.0		51.9	52.4		53.9	53.3		43.3	39.3	
Year of first RRT (%)			0.34			0.001			0.40			0.76			0.011
1987-1990	19.4	16.6		19.4	13.0		18.6	16.7		16.7	14.4		12.4	14.8	
1991-1994	24.1	27.1		24.3	27.5		24.2	26.3		22.1	25.1		18.0	20.6	
1995-1998	28.5	25.8		28.1	30.1		28.0	27.3		27.6	28.5		30.3	30.5	
1999-2002	28.0	30.5		28.2	29.4		29.2	29.7		33.6	32.0		39.3	34.1	

N = number of patients, yr = year, GN = glomerulonephritis, HT = hypertension, RVD = renovascular disease, DM = diabetes mellitus, RRT = renal replacement therapy, HD = hemodialysis, PD = peritoneal dialysis, p = p-value

**Table 3:** Multivariable-adjusted and propensity score-stratified models without interaction variables

	Multivariable-adjusted model		Propensity score-stratified model	
	**HR**	**(95% CI)**	**HR**	**(95% CI)**
Age (per yr)	1.05	(1.05; 1.05)	-	-
Female vs male gender	0.89	(0.85; 0.93)	-	-
Primary renal disease vs GN*			-	-
HT	1.25	(1.13; 1.38)	-	-
RVD	1.74	(1.57; 1.92)	-	-
DM	2.24	(2.05; 2.46)	-	-
Other	1.33	(1.22; 1.44)	-	-
Year of first RRT (per yr)	0.99	(0.98; 1.00)	-	-
Dialysis center	0.13 - 1.61^#^		-	-
Peritoneal vs hemodialysis	0.99	(0.94; 1.05)	0.97	(0.92; 1.03)

* Compared with GN as reference group.

^# ^Range of HRs, we did not provide 95%CIs for center because of the large number of estimates.

yr = year, GN = glomerulonephritis, HT = hypertension, RVD = renovascular disease, DM = diabetes mellitus, RRT = renal replacement therapy, HR = hazard ratio

#### Multivariable regression analysis

In the unadjusted Cox model, patients receiving PD had a 30% lower mortality compared with HD patients (HR = 0.70; 95%CI: 0.67-0.74; p < 0.001). The coefficients of all other univariable models were also statistically significant (p-values ranging from < 0.001 to 0.02), both in the overall population and in the HD and PD groups separately. The coefficient for the year of starting RRT was not significant in the total population, because with increasing year of start of RRT, the relative risk of dying increased for HD patients and decreased for PD patients.

In contrast to the univariable model, the multivariable Cox model, adjusted for main effects of age, gender, primary renal disease, year of first RRT and treatment center but without interaction terms revealed that mortality of PD patients and HD patients did not differ significantly (Table 3). The HR of PD compared with HD patients was 0.99 (95%CI: 0.94-1.05), which did not differ from the relative risk estimated by the propensity score-stratified model (HR 0.97; 95%CI 0.92-1.02). Note however that the propensity score model involved only one covariate as opposed to nine in the multivariable Cox model.

#### Exploration of effect modification

The constructed Cox models did not consider the possibility of effect modifiers on outcome. When tested in the multivariable model, four interaction variables were statistically significant: two with modality (age by modality (HD or PD) and diabetes as the primary cause of renal disease (PRD-DM) by modality), and two other interaction variables (age by PRD-DM and gender by PRD-DM). After entering these interaction variables into both the propensity score-stratified and the multivariable-adjusted model (Table 4), the hazard ratios of dialysis modality and all interaction variables with dialysis modality were statistically significant in both models. As before, the results from the propensity score-stratified and multivariable-adjusted models were essentially identical. The propensity score-stratified model, however, included almost the same number of variables as the multivariable-adjusted model. The only two additional variables in this multivariable-adjusted model were year of first RRT and dialysis center. Hazard ratios for the combinations of the interaction variables as estimated by the multivariable-adjusted model are presented in Table 5. They show a relative survival benefit of PD compared with HD that diminishes with increasing age and in the presence of diabetes. Since the proportionality assumption was not satisfied, time-stratified analyses are presented. The clinical issues associated with these findings are discussed more in depth elsewhere [11].

**Table 4:** Multivariable-adjusted and propensity score-stratified models with interaction variables

	Multivariable-adjusted model		Propensity score-stratified model	
	HR	(95% CI)	HR	(95% CI)
Age (per yr)	1.05	(1.04; 1.05)	1.05	(1.04; 1.05)
Female vs male gender	0.87	(0.83; 0.91)	0.87	(0.83; 0.91)
Primary renal disease vs GN*	-	-	-	-
HT	1.22	(1.10; 1.35)	-	-
RVD	1.68	(1.51; 1.85)	-	-
DM	5.65	(3.95; 8.09)	5.36	(3.73; 7.70)
Other	1.31	(1.21; 1.42)	-	-
Year of first RRT (per yr)	0.99	(0.99; 1.00)	-	-
Dialysis center	0.13 - 1.61^#^	^†^	-	-
Peritoneal vs hemodialysis	0.43	(0.32; 0.57)	0.44	(0.32; 0.60)
Age × Dialysis modality	1.01	(1.01; 1.02)	1.01	(1.01; 1.02)
DM × Dialysis modality	1.22	(1.08; 1.38)	1.23	(1.08; 1.40)
Age × DM	0.98	(0.98; 0.99)	0.98	(0.97; 0.99)
Gender × DM	1.20	(1.07; 1.34)	1.20	(1.07; 1.35)

* Compared with GN as reference group.

# Range of HRs.

† Not presented because of range of HRs.

yr = year, GN = glomerulonephritis, HT = hypertension, RVD = renovascular disease, DM = diabetes mellitus, RRT = renal replacement therapy, HR = hazard ratio

**Table 5:** Associations between dialysis modality and mortality for various combinations of the effect modifiers

		Hazard ratios of peritoneal dialysis vs. hemodialysis (95% Confidence Intervals)*		
		
		>3-6 months^**#**^	>6-15 months^**#**^	>15 months^**#**^
Age	DM			
			
40	No	0.26 (0.17; 0.41)	0.51 (0.39; 0.68)	0.86 (0.74; 1.00)
40	Yes	0.40 (0.23; 0.68)	0.59 (0.44; 0.81)	1.06 (0.88; 1.26)
50	No	0.35 (0.25; 0.48)	0.62 (0.51; 0.76)	0.95 (0.85; 1.05)
50	Yes	0.53 (0.34; 0.83)	0.72 (0.56; 0.93)	1.17 (1.00; 1.35)
60	No	0.46 (0.37; 0.58)	0.75 (0.65; 0.87)	1.05 (0.97; 1.13)
60	Yes	0.71 (0.48; 1.04)	0.87 (0.71; 1.09)	1.29 (1.12; 1.48)
70	No	0.62 (0.50; 0.76)	0.92 (0.80; 1.05)	1.16 (1.07; 1.25)
70	Yes	0.95 (0.64; 1.39)	1.07 (0.85; 1.33)	1.42 (1.23; 1.65)

* From models including age, gender, primary renal disease, year of start of RRT, center and the interaction terms age by modality, diabetes by modality, age by diabetes and gender by diabetes.

DM = diabetes as primary renal disease

# Models stratified for time on dialysis, >3-6 months, >6-15 months, and >15 months.

## Conclusion and Discussion

In this study of nearly all patients who initiated chronic dialysis treatment between 1987 and 2002 in The Netherlands, we developed a propensity score that fulfilled accepted quality criteria: it showed a reasonable fit, had a good c-statistic, calibrated well and balanced the covariates. The Cox model that solely stratified on propensity score yielded essentially identical effect estimates of PD versus HD mortality compared with the multivariable-adjusted model while having the advantage of containing only one as opposed to nine covariates, thus being more parsimonious. When excluding interaction terms, dialysis modality was not an independent predictor of mortality in either model, but both models were misspecified, because effect modification was present. After introducing both interaction terms and all corresponding main effect variables to account for effect modification, the propensity score-stratified model contained almost the same number of covariates as the multivariable-adjusted model. When the models included interaction variables, all covariates remained independent predictors, but now dialysis modality besides its interaction variables became statistically significant. Supporting our findings, the identified effect modifiers, age and diabetes as primary renal disease, correspond to those found in previous studies [12-15].

Our study informs the discussion of the utility of propensity score in outcomes research. In theory, the use of propensity score-stratified modeling may allow for a more straightforward estimation of the relative mortality risk in comparison with multivariable-adjusted modeling. However, our study shows that neglecting effect modification in propensity score-stratified models may lead to erroneous conclusions. Incorporating effect modification however removes the direct interpretability of the main treatment effect one wishes to estimate, thereby limiting one benefit of using a propensity score. Still, Sturmer and colleagues [16] suggest that the propensity score-adjustment approach may yet have an advantage over traditional methods when effect modification is present, because it allows for a summary effect size across all strata of the effect modifiers. This can be relevant in pharmacoepidemiology, to estimate how a total population might benefit from a particular drug. However in the setting of end-stage renal disease, the assignment of a patient to a specific dialysis modality should be tailored to a patient's specific pretreatment characteristics and should not be determined by the summary effect size across all strata of the effect modifiers.

Other studies that compared propensity score-stratified versus multivariable-adjusted modeling have been reviewed by Shah and colleagues [4] and Sturmer and colleagues [1]. Similar to our findings, both studies concluded that propensity score-stratified modeling rarely led to a different result compared with multivariable-adjusted modeling. Choosing which method may depend on the quantity of data. Cepeda and colleagues report from their simulation study that with eight or more outcomes per confounder, the multivariable-adjusted logistic regression model showed better precision [17]. However, with fewer than eight events per confounder, propensity score-stratified modeling performed better. Furthermore, the choice also depends on the research question, as suggested by Kurth and colleagues [18]. They showed that when there is a non-uniform treatment effect, different adjustment methods can result in divergent results, which may all be correct but depend on the research question implied by the adjustment method. Propensity score, as opposed to traditional multivariate modeling, enables a direct estimation of comparability of the treatment groups by assessing the covariate balance between groups [4].

For the propensity score-adjustment approach, there are no accepted rules for construction and evaluation of the propensity score model. Evaluation of a propensity score model often consists of assessing discrimination with the c-statistic and calibration with goodness-of-fit tests. However, Weitzen and colleagues in their simulation study showed that neither the c-statistic nor the Hosmer-Lemeshow goodness-of-fit test was sensitive to omission of an important confounder from the propensity score model [19].

Failure to include important confounders can lead to biased estimates of the treatment effect [20]. Austin and colleagues reported that propensity scores estimated on administrative data might not balance all clinical characteristics [21]. This could be particularly relevant to our study, because the RENINE database is administrative and does not contain clinical data, in particular co-morbidity data. Information on primary renal diagnosis however is available with the most important co-morbid condition, diabetes, likely well-represented because of the strong correlation between diabetes and diabetes as primary renal disease (PRD-DM). Further, Weitzen and colleagues [19] report that omitting a confounder in a propensity model has little effect on the treatment effect estimate. This could imply that the propensity score is fairly robust to unobserved confounders if, as also reported by Rosenbaum [3], at least some of the key variables that explain treatment assignment are included in the score. Moreover, omitting a confounder also leads to biased estimates when using a multivariable-adjusted model.

To summarize: if propensity score models are constructed well and no important confounders are missing, a treatment effect with a reliable significance level can usually be estimated, with the advantage of a more parsimonious outcome model and the advantage of assessing covariate balance between treatment groups explicitly. When the outcome is rare, propensity score-adjustment yields effect size estimates with a higher precision. Reviews of studies applying propensity score-adjustment methods have shown however, that propensity score-stratified modeling was often not implemented or reported appropriately [1,4,10]. Researchers should carefully assess whether propensity score-adjustment methods are appropriate for their specific situation.

From our study, we conclude that although the propensity score performed well, it did not alter the treatment effect in the outcome model and lost its advantage of parsimony because effect modification was present. Thus, using a model of mortality of patients on renal replacement therapy as a special case study, our study contributes to the growing literature supporting the comparability of traditional multivariable regression and propensity score methods unless sample size is small and outcome is rare.

## Abbreviations

ERA-EDTA: European Renal Association-European Dialysis and Transplantation Association; ESRD: end-stage renal disease; HD: hemodialysis; HR: hazard ratio; PD: peritoneal dialysis; PRD-DM: primary renal disease = diabetes mellitus; PRD-GN: primary renal disease: glomerulonephritis; PRD-HT: primary renal disease = hypertension; PRD-RVD: primary renal disease = renovascular disease; PRD-OTH: primary renal disease = other renal diagnoses; RENINE: Dutch End Stage Renal Disease Registry; RRT: renal replacement therapy; SD: standard deviation.

## Competing interests

The authors declare that they have no competing interests.

## Authors' contributions

YSL contributed to the design of the study, the analysis and interpretation of data, and the drafting of the manuscript, JBW and MGMH contributed to the design of the study, the analysis and interpretation of data, and critically revised the manuscript, FThC contributed through the acquisition of the data and critical revision of the manuscript, WCW contributed to the conception and design of the study, the analysis and interpretation of data, and critically revised the manuscript. All authors read and approved the final manuscript.
